# Formulation and Evaluation of Licorice-Extract-Enhanced Chitosan, PVA, and Gelatin-Derived Hydrogels for Wound Dressing

**DOI:** 10.3390/bioengineering12050439

**Published:** 2025-04-23

**Authors:** Maria Mujahid, Muhammad Zubair, Asma Yaqoob, Sohail Shahzad, Aman Ullah

**Affiliations:** 1 Department of Chemistry, University of Sahiwal, Sahiwal 57000, Pakistan; mariamujahidch@gmail.com; 2 Lipid Chemistry Utilization Lab, Department of Agricultural, Food & Nutritional Science, University of Albert, Edmonton, AB T6G 2P5, Canada; mzubair1@ualberta.ca; 3 Institute of Biochemistry, Biotechnology and Bioinformatics, The Islamia University of Bahawalpur, Rabia Hall Rd, Bahawalpur 63100, Pakistan; asma.yaqoob@iub.edu.pk

**Keywords:** chitosan, PVA, freeze gelation, gelatin, wound dressing, antibacterial

## Abstract

Wound infections remain a significant clinical challenge, impeding healing and causing deterioration. Recently, multifunctional hydrogel dressings have gained interest as an effective treatment to treat infections efficiently and enhance wound recovery. The present research is focused on the development of composite hydrogels comprising chitosan (CS), polyvinyl alcohol (PVA), gelatin (GEL) and licorice extract (LE), using the freeze gelation technique. The resulting composite hydrogels of CS/PVA/GEL incorporating LE were characterized by FTIR, XRD and SEM. FTIR analysis confirmed the presence of specific functional groups within the molecules. XRD exhibited the amorphous nature of hydrogels. SEM analysis revealed that increasing the CS ratio in hydrogels created a more porous structure with a smaller pore size. All the hydrogels demonstrated oxygen permeability, which is crucial for the healing process. Among the synthesized hydrogels, MM-2 containing PVA (20 mL) and LE (4 mL) demonstrated superior performance with a water retention capacity of 440% and moisture content of 91%. This exceptional result can be attributed to the higher proportion of PVA and the material’s porous structure, which enhances its hydrophilic properties. The synthesized hydrogels showed good antibacterial potential against three selected strains of bacteria including *Bacillus subtilis* (*B. subtilis*), *Staphylococcus aureus* (*S. aureus*) and *Escherichia coli* (*E. coli*). The hydrogels’ cytotoxicity levels were assessed through hemolysis assay and the results demonstrated that all hydrogels were non-toxic. The hydrolytic breakdown revealed that the interconnected hydrogels with licorice components exhibited slow degradation, making them more appropriate for long-term wound treatment. Specifically, MM-4 demonstrated a 74% degradation rate and displayed 75% antioxidant activity, indicating its potential effectiveness for chronic wound applications. These characteristics of synthesized CS/PVA/GEL/LE-derived hydrogels suggest their potential use as a promising candidate for wound care applications.

## 1. Introduction

Skin, comprising 15% of total body mass, serves as the primary defense mechanism for the human body [1,2,3]. This vital organ faces considerable stress from prolonged contact with various external factors, rendering it vulnerable to a wide array of stimuli [4,5]. When the skin’s protective barrier is compromised, such as through wound formation, it becomes a potential source for numerous health issues [6]. Thus, wound coverage is essential for protection and plays a crucial role in the healing process by acting as a temporary skin substitute [7,8]. It is crucial to provide suitable, non-toxic [9] wound care solutions with antibacterial properties for faster recovery of wounds [10]. Recent scientific and technological advancements have enabled the development of various new techniques and materials for the formulation of wound dressings because traditional methods cannot be used for chronic wounds or severe injuries [11].

Hydrogels are useful in the majority of injuries, but for significant skin injuries, a wound dressing that mimics the extracellular matrix (ECM) is necessary. These wound dressings have properties comparable to skin tissues in order to enable cell adhesion, migration, proliferation, and tissue creation [12]. The hydrogels fabricated by freeze gelation methodology encompass various advantages, including more control over the hydrogel’s structure and characteristics, decreased toxicity, and improved safety compared to chemical or thermal gelation techniques [13,14]. Natural and synthetic polymers are being extensively used nowadays for the formulation of porous hydrogels with their beneficial characteristics of biodegradability, biocompatibility, elasticity, non-toxicity, inherent antimicrobial potential, mechanical strength, and cell adhesion [13,15,16,17].

Chitosan (CS), a naturally occurring cationic polysaccharide, exhibits biological, chemical, and physical properties that make it an ideal material for use in wound dressings and other biomedical applications. CS has hemostatic and intrinsic antibacterial potential, and it also reduces inflammation and scar formation. Moreover, CS also boosts the body’s endogenous hyaluronic acid at the wound site, which promotes wound healing [18].

Polyvinyl alcohol (PVA) is a hydrophilic and synthetic polymer. It is an excellent material for various biomedical applications due to its biocompatible, biodegradable, flexible, non-toxic, and non-carcinogenic nature [19,20]. Its better mechanical strength and transparent nature also make PVA popular for use in the formulation of wound-healing materials [21].

Gelatin (GEL), a biopolymer produced by partially hydrolyzing or thermally denaturing collagen, has found extensive applications in tissue engineering and regenerative medicine due to its high biodegradability and biocompatibility [22]. It swiftly concentrates huge volumes of red blood cells and platelets by quickly absorbing blood and increasing the formation of clots in the blood, hence plugging wounds and imposing efficient hemostasis [23,24].

Hydrogels prepared via chemical crosslinking methods sometimes require higher concentrations of crosslinkers that may modify hydrogel properties and also introduce biosafety concerns. Thus, natural plant-based components are used as a substitute for chemical crosslinkers to reduce biosafety and toxicity issues [25,26,27].

**Figure 1:**
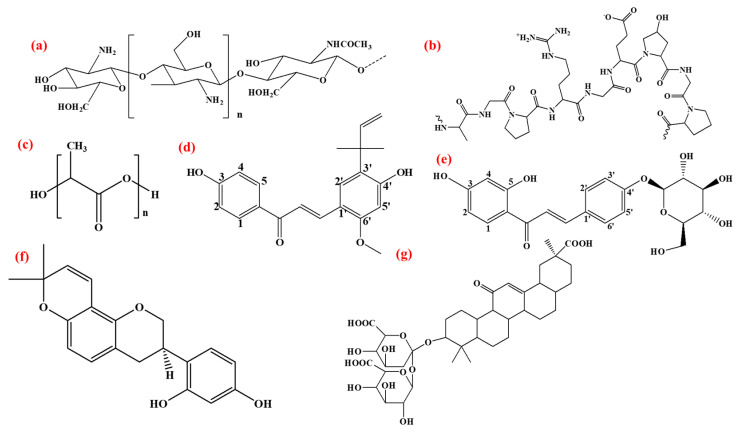
Structure of (**a**) chitosan, (**b**) gelation, (**c**) PVA, and active components of licorice: (**d**) licochalcone A, (**e**) isoliquirtin, (**f**) glabridin, (**g**) glycyrrhizin.

Licorice root (*Glycyrrhiza glabra*) has been in use by humans for around 4000 years [28]. Glycyrrhizic acid is a triterpenoid saponin and the main active compound of licorice root, which imparts a sweet taste and is responsible for other therapeutic effects due to its antiviral, anti-inflammatory, and antibacterial potential [29]. The licorice root also contains flavonoids, polyphenols, and other bioactive compounds such as chalcones, triterpenes, isoflavonoids, and saponins, as shown in Figure 1 [30,31,32]. These natural compounds have the potential to reduce excessive inflammation during wound healing and also reduce oxidative stress by neutralizing the free radicals at the wound site [33,34]. Table 1 shows the advantages and limitations of previous studies that urge the need for new research combinations. Herein, we have developed composite CS/PVA/GEL-based hydrogels, incorporated with LE for wound dressing applications.

## 2. Materials, Synthesis, and Characterization

### 2.1. Chemicals

All chemicals and solvents were used exactly as they were purchased from suppliers or redistilled as required. CS with a degree of deacetylation greater than 90% and molecular mass ranging from 100 to 200 kDa was purchased from Macklin chemicals (Shanghai, China). PVA (Mw: 72,000, degree of hydrolysis 98%), gelatin, and sodium hydroxide (NaOH) were purchased from Merck (Darmstadt, Germany). Glacial acetic acid (CH_3_COOH) was purchased from AnalaR BDH Laboratory Supplies (Dorset, UK). Licorice plant roots were purchased from a local market in Sahiwal, Pakistan. The entire experiment was conducted using deionized water. All other reagents and solvents were of analytical grade and were utilized directly.

#### 2.1.1. Preparation of Licorice Extract (LE)

Licorice plant roots (*Glycyrrhiza glabra*) were cleaned and subsequently ground. Then 10% *w*/*v* aqueous solution of these was prepared by heating the mixture at 60 °C at 600 rpm for 3 h. The resulting solution was filtered using Whatman filter paper, and the filtrate was preserved at 4 °C for later use in the fabrication of hydrogels.

#### 2.1.2. Synthesis of CS/PVA/GEL/LE Hydrogels

**Figure 2:**
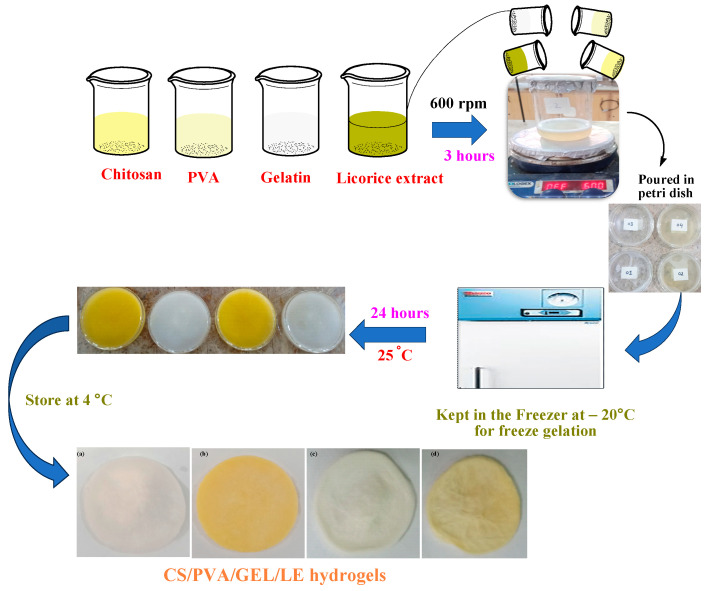
Schematic representation of the preparation of CS-PVA-GEL-LE hydrogels.

CS/PVA/GEL/LE composite hydrogels were fabricated through the freeze gelation method, as described by Khan et al., with some modifications [38]. For the fabrication of hydrogels, a 1% chitosan solution was made by dissolving 1 g of chitosan in 1% acetic acid solution by stirring at 600 rpm for 24 h at room temperature. A 5% PVA aqueous solution was prepared by dissolving PVA at 80 °C at 600 rpm for 3 h, while a 2% aqueous gelatin solution was prepared by dissolving gelatin at 105 rpm at 40 °C for 1 h. Various volume ratios of CS, PVA, GEL, and LE solutions were fabricated as outlined in Table 2. The solutions were mixed and homogenized by stirring at 600 rpm for at least 3 h. As in hydrogel MM-1, 20 mL of CS, 20 mL of PVA, and 3 mL of GEL solutions were mixed. While in hydrogel MM-2, along with all the ratios of MM-1, 4 mL of LE was added. In hydrogel MM-3, 28 mL of CS, 12 mL of PVA, and 3 mL of GEL were used. Similarly, in hydrogel MM-4, 4 mL of LE was added with all the proportions of MM-3. Following homogenization, the blended solutions were transferred into Petri plates and kept in the refrigerator for 72 h at −20 °C. These hydrogels were then immersed in pre-cooled 3 M NaOH/ethanol solution and then refrigerated again for 48 h at −20 °C. The hydrogels were washed with 50% ethanol followed by absolute ethanol. Finally, the hydrogels were rinsed with distilled water until they were neutralized. The synthesized hydrogels were then allowed to dry at 25 °C and stored at 4 °C until required. The hydrogels were coded as MM-1, MM-2, MM-3, and MM-4. The detailed synthesis of hydrogels has also been illustrated in Figure 2.

### 2.2. Physical and Structural Characterization of Hydrogels

The physicochemical interactions amongst functional groups of various components of hydrogels were evaluated by Fourier transform infrared (FTIR) Spectroscopy (Thermo Nicolet 6700P, Waltham, MA, USA). A photo acoustic cell with a carbon background and helium purging was used to capture FTIR spectra at room temperature. The resolution was 8 cm^−1^, and there were 256 scan numbers within the range of 4000 and 400 cm^−1^.

Hydrogel thickness was measured in millimeters (mm) using a digital screw gauge (Haidian District, Beijing, China) with 0–25 mm/0.01 mm resolution range. Five arbitrary measurements of the hydrogel’s surface, including center, surroundings, and remaining positions, were performed, and the arithmetic mean was computed.

The physical appearance, color, and homogeneity of the hydrogels were assessed using images captured with a Redmi Note-11 Pro mobile camera (108 megapixels, 1× magnification). A scanning electron microscope (SEM) model EmCrafts Cube II tabletop (EmCrafts Co., Ltd., Gwangju-si, Republic of Korea) was used to examine the surface and cross-sectional morphology of CS/PVA/GEL/LE hydrogels. The samples were sputter-coated, positioned on SEM holders, and then imaged using an accelerating voltage of 10 kV. The images were scanned at different magnification levels. From the resulting SEM images, the average diameter of 20 pores was measured using ImageJ software (1.46r). While measuring the pore diameter, the deeper visible pores on the surface of hydrogels were also taken into account.

X-ray diffractometer (XRD), D8 advance (Bruker, Billerica, MA, USA), model cube 10, was used to analyze the crystalline/amorphous nature of CS/PVA/GEL/LE hydrogels. The 2θ range of 10° to 70° was used to scan all hydrogels at a scanning speed of 17.22 s, with a 0.09 step size.

### 2.3. Measurement of Total Phenolic and Flavonoid Contents

The total phenolic contents (TPCs) of LE were evaluated using the Folin–Ciocalteu colorimetric method as described in the literature [39]. First, 200 µL of LE (100 mg/mL) was taken in a test tube, and then 1 mL of Folin–Ciocalteu reagent was added and the solutions were mixed thoroughly. Then 800 µL of 7.5% Na_2_CO_3_ solution was added into mixture and the mixture was left at 25 °C for 2 h. Finally, the mixture was shaken well and its absorbance was measured at 765 nm using a STA-8100ST UV/Vis spectrophotometer (Van Nuys, Los Angeles, CA, USA). Gallic acid was used as a standard and described as Gallic acid equivalent (GAE) per dry matter [40]. The total flavonoid contents (TFCs) of LE at a concentration of 100 mg/mL were evaluated using a methodology described in the literature with slight changes [41]. Fresh reaction reagents were prepared, including 10% Al_2_Cl_3_, 5% NaNO_2_, and 1 M NaOH. Additionally, 1 mL of LE was combined with 400 µL of 5% NaNO_2_ in a test tube and mixed thoroughly. Subsequently, 6 mL of distilled water was introduced, and the mixture was incubated for 10 min. Then 700 µL of 10% Al_2_Cl_3_ and 3 mL of 1 M NaOH were added. The TFCs amount was calculated as catechins equivalent per dry matter by taking absorbance at 510 nm.

### 2.4. Physical and Functional Assessments of the Hydrogels

Winkler’s methodology (dissolved oxygen in mg/mL) was used to determine the O_2_ permeability of CS-PVA-GEL-LE hydrogels. For this, the flasks were filled with distilled water up to the mark, then sealed using sealant with hydrogels as a cap.

A closed flask, completely filled with water, was used as a negative control, and an open flask served as a positive control. The dissolved oxygen in the flasks was measured using an oximeter, after a 24 h period [42].

The hydrogel’s moisture content percentage (MC%) was evaluated by the loss on drying methodology. The hydrogels were meticulously divided into tiny pieces and were precisely weighed. Then, the hydrogels were completely dried in an oven at 100 °C until a constant weight was achieved. Equation (1) was applied to calculate the moisture content percentage (MC%) of hydrogels.

(1) $$MC\%=\frac{M_{w}-M_{d}}{M_{w}}\times 100$$

M_W_ stands for preliminary weight of hydrogels conditioned at 70% relative humidity to moisture equilibrium, whereas *M_d_* represents the dried weight of hydrogels. The experiment was run three times, and average results were used in subsequent calculations [43].

The hydrogel solution absorption capability was determined in phosphate-buffered saline (PBS) solution (pH = 7.4). Each hydrogel sample was cut into small pieces, and its dried weight was recorded. The hydrogels were then immersed in PBS solution at room temperature and removed after every 10 min. The weights of solution-absorbed hydrogels were noted after removing their surface water with a tissue, until no further increase in their weights was observed [44]. Equation (2) was used to determine the percentage of solution absorption capacity for each hydrogel.

(2) $$S=\frac{M_{S}-M_{d}}{M_{d}}\times 100$$

Here, M_s_ represents the solution absorbed weight and M_d_ represents the dried weight.

The water retention capacity of the prepared hydrogels was evaluated by taking small pieces of the hydrogels. The hydrogel pieces were weighed accurately and then immersed in deionized water at room temperature. The hydrogels were removed after every 30 min to check their weight gain and then immersed back in water until they absorbed maximum and no further gain in weight was noted. The hydrogels were then dried at room temperature for 24 h, and their final weight was measured [45]. Equation (3) was used to measure the water retention capacity percentage of synthesized hydrogels.

(3) $$Water\;retention\;capacity\;(\%)=\frac{W_{t}}{W_{o}}\times 100$$

Here, W_o_ donates the initial weight and W_t_ is the hydrogel’s water retained weight.

### 2.5. Antibacterial Activities of Synthesized Hydrogels

Antibacterial activities of composite CH/PVA/GEL hydrogels incorporating LE were evaluated against *Bacillus subtilis* (*B. subtilis*), *Staphylococcus aureus* (*S. aureus*), and *Escherichia coli* (*E. coli*) using the disc diffusion method. The procedure followed the standard protocol outlined by Bauer et al. (1966) [46]. First, 100 µL suspensions of three bacterial strains, containing 10^7^ colony-forming units (CFU)/mL of bacterial cells, were cultured for 8 h at 37 °C in nutrient broth (Oxoid, UK). Bacterial culture was then evenly spread on agar plates using a sterile swab. Then the Petri plates were dried for 15 min. The hydrogels were cut and applied to the Petri plates. Each Petri dish contains four hydrogel discs placed at about equidistance with one plate for each bacterial strain acting as a positive control, which is prepared from a standard commercial antibiotic ciprofloxacin (10 µg/mL). Finally, Petri dishes were placed at 4 °C for 2 h and incubated at 37 °C for 24 h. The inhibition zone was measured using calipers and noted. The assay was conducted in triplicate to ensure reliability. The antibacterial potential was assessed by computing the diameter of inhibition zones in millimeters exhibited by hydrogels and comparing it with the positive control [47].

### 2.6. Anti-Hemolytic/Cytotoxicity Analysis of the Hydrogels

The anti-hemolytic/cytotoxicity analysis of all the fabricated hydrogels was evaluated using previously reported methods (workflow is mentioned in Figure S2) [48,49]. Fresh bovine blood (3 mL) was obtained and centrifuged at 1000 rpm for 10 min. After discarding the plasma, the remaining red blood cells (RBCs) underwent three washing cycles with cold (4 °C) sterile isotonic PBS solution (pH 7.4). RBC concentration was maintained at 10^8^ cells per mL for each test. Hydrogel samples (10 mg) were immersed in 1 mL of distilled water, and then 20 μL of the resulting solution was combined with human erythrocytes (10^8^ cells/mL) separately. The hydrogel samples were incubated at 37 °C for 45 min, with agitation after 10 min. Subsequently, the hydrogel samples were cooled on ice for 5 to 10 min and centrifuged again at 1000 rpm for 10 min. From each test tube, 100 µL of supernatant was extracted and diluted tenfold with chilled (4 °C) PBS solution (pH 7.4). A 0.1% *v*/*v* Triton X-100 solution served as the positive control, while PBS solution (pH 7.4) was used as the negative control. Absorbance measurements were taken at 576 nm using a UV–vis spectrophotometer (UV–VIS Spectrophotometer, STA-8100ST Stalwart-Van Nuys, CA, USA). The percentage of RBC lysis was calculated for all hydrogel samples and controls.

### 2.7. Biodegradation Studies

Biodegradability of fabricated hydrogels was assessed in PBS solution of pH 7.4. For this assay, small pieces of hydrogels were cut and their weight (W_1_) was noted. Then, the hydrogels were soaked in PBS solution for 1 to 42 days at a temperature of 35 ± 2 °C. At pre-determined intervals of seven days, the samples were removed, dried at 37 °C, and their weight (W_2_) was measured [50]. The percent degradation was then calculated using the following Equation (4).

(4) $$Degradation\left(\%\right)=\left[\frac{W_{2}}{W_{1}}\right]\times 100$$

### 2.8. Anti-Oxidant Properties

The extent of antioxidant activity is determined by the plant components’ ability to scavenge the 2,2-diphenylpicrylhydrazyl (DPPH) radical. In this procedure, the plant extract was prepared by combining 20 mg of hydrogel with 3 mL of distilled water. Subsequently, 1 mL of this mixture was combined with 0.2 mL of methanolic DPPH solution (1 mM). The plant extract and DPPH solutions were then blended and stored in darkness at ambient temperature for approximately 40 min. Lastly, the absorbance of the combined plant extract and DPPH solution was measured at 517 nm against a blank solution using a UV–visible spectrophotometer [51]. The process was repeated three times and the % of DPPH radical scavenging activity was calculated using the following formula:

$$\mathrm{Scavenging}\;\mathrm{activity}\;(\mathrm{SA}\%)\;=\frac{Acontrol-Asample}{Acontrol}\times 100$$

where A_control_ shows the absorbance of the control, which is the DPPH solution, and A_sample_ stands for the absorbance of the plant extract by DPPH.

### 2.9. Statistical Analysis

In each measurement of the physiochemical parameters of the hydrogels, at least three measurements were used. The mean ± standard deviation was used to express the results with *p* < 0.05, significance = 1. Differences between groups were tested by one-way ANOVA with the Tukey test using Origin Pro 2016.

## 3. Results and Discussion

### 3.1. Structural and Morphological Analysis

**Figure 3:**
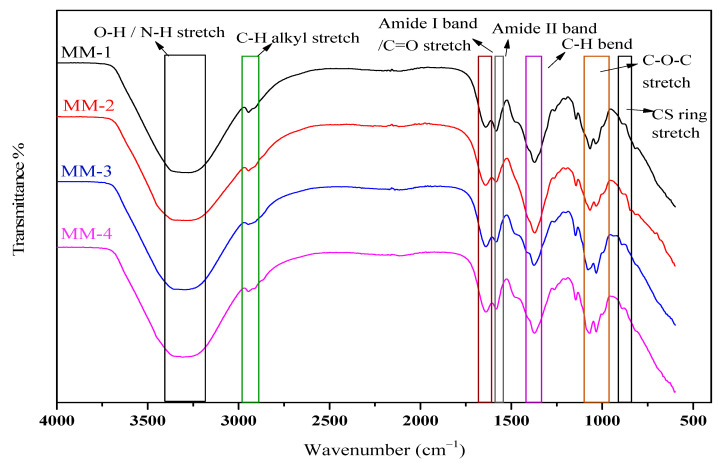

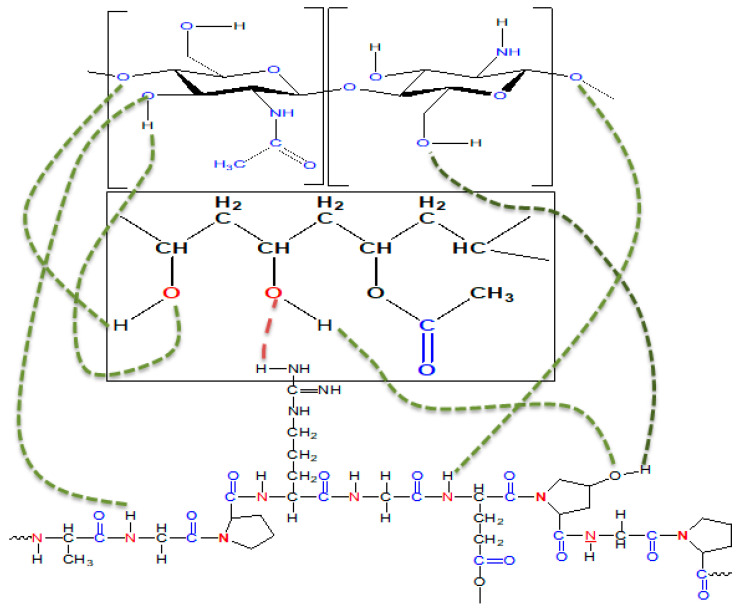
FTIR analysis and interaction among polymers of all the synthesized hydrogels.

FTIR was performed to examine the intermolecular interactions between the different functional groups of individual components found in synthesized hydrogels. All the synthesized hydrogels’ FTIR spectra have been displayed in Figure 3, which shows a relatively broad peak between 3370 cm^−1^ and 3220 cm^−1^ due to overlapped stretching frequencies of the hydroxyl (O-H) and amino (N-H) groups. Between 2960 cm^−1^ and 2910 cm^−1^, both symmetric and asymmetric stretching frequencies of the alkyl C-H groups were observed. Peaks of amide I and amide II bands appeared at 1669 cm^−1^ and 1654 cm^−1^, respectively [52]. A small peak at 1600 cm^−1^ represents the carbonyl (C=O) stretch, which is present in chitosan’s structure due to non-deacetylated groups [53]. A sharp peak between 1066 cm^−1^ and 1018 cm^−1^ indicates a stretching vibration of C-O-C in pure chitosan. Chitosan’s ring stretching vibrations appeared in the fingerprint region at 895 cm^−1^ [54]. It has also been observed that N-H/O-H stretching vibration peaks became broader and lost their sharpness in MM-2 and MM-4 hydrogels as compared to MM-1 and MM-3 hydrogels spectra. This might be due to the addition of LE and the presence of certain hydroxyl groups of polyphenols in LE. Similarly, slight changes in certain other peak frequencies in MM-1 and MM-3 (without LE) as compared to MM-2 and MM-4 (incorporating LE) were also noticed.

**Figure 4:**
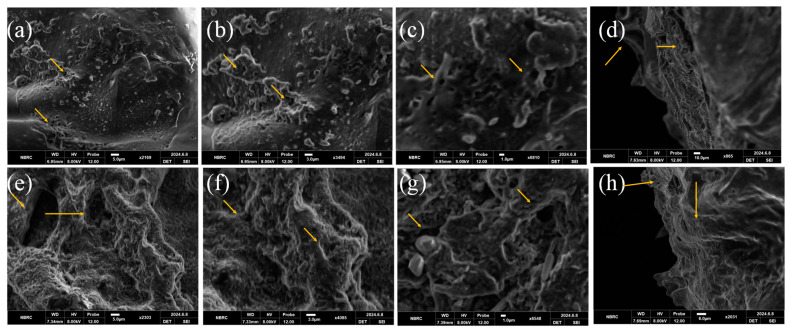
Surface SEM images of MM-1 (**a**–**c**), while cross-sectional image of MM-1 (**d**) and surface images of MM-2 (**e**–**g**), while cross-sectional image of MM-2 (**h**); represents pores (shown with arrows) within hydrogels. The pore size of MM-1 is 12.92 ± 5 µm and MM-2 is 17 ± 5 µm.

**Figure 5:**
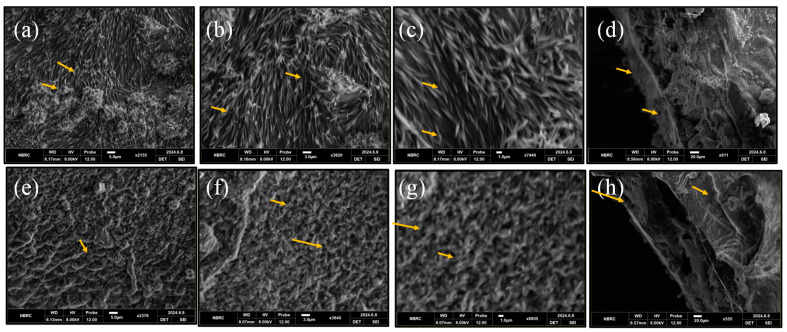
Surface SEM images of MM-3 (**a**–**c**), while cross-sectional image of MM-3 (**d**) and surface images of MM-4 (**e**–**g**), while cross-sectional image of MM-4 (**h**); represents porous network (shown with arrows) within hydrogels. The pore size of MM-3 is 18.83 ± 5 µm and MM-4 is 25.06 ± 5 µm.

The hydrogel’s surface and cross-sectional morphology were revealed by SEM images as depicted in Figure 4 and Figure 5. The MM-1 hydrogel (composed of CS/PVA/GEL) has exhibited irregular surface morphology with uneven portions, accompanied by a few pores and certain white color crystalline parts as depicted by images of various magnifications in Figure 4. This morphology was produced due to intermolecular polymer network interactions amongst CS, PVA, and GE during vigorous blending, freezing, and gelation processes. Meanwhile, SEM micrographs of MM-2 hydrogel showed their sponge-like structure, higher porosity, and better homogeneity, which was created by the addition of LE into CS/PVA/GE composite hydrogels. Thus, it can be inferred that the addition of LE into equal volume ratios of CS and PVA has produced a profound effect in the morphology of hydrogels.

Similarly, the SEM images of MM-3 and MM-4 hydrogels shown in Figure 5 are quite different from the SEM images of MM-1 and MM-2. The SEM images of MM-3 and MM-4 hydrogels showed a highly porous structure as compared to MM-1 and MM-2. This might be due to a change in CS and PVA ratios, as we increased the CS volume ratio and reduced the PVA volume ratio in MM-3 and MM-4 hydrogels. The MM-3 hydrogel showed a highly fibrous and porous structure, which offers cell migration and adhesion, crucial for tissue regeneration throughout the healing process. Additionally, the effect of the incorporation of LE in MM-4 hydrogel was also clearly evident from SEM images. The morphology of MM-4 hydrogel was observed to be highly porous, homogeneous, interconnected, and spongy, as compared to MM-3 (which appeared to be fibrous), which might be shaped by the interactions of active LE components with CS, PVA, and GE during blending and freeze gelation. The porous characteristics of hydrogels are associated with their ability to provide a continuous moist surface at the wound site. Similarly, when porous hydrogels are placed at the wound site, active ingredients from these hydrogels gradually release for extended periods which prevents bacterial invasion and supports healing processes [55]. Our results are also supported by literature studies, which also showed that increasing the CS ratio in CS/PVA hydrogels also increases their porous network [56]. The mean size of pores was observed to be increased as the CS volume ratio was enhanced in hydrogels. The three-dimensional (3D) porous structure of hydrogels makes it easier for blood and tissue fluid to be absorbed and for cellular nutrients to be transported, which promotes hemostasis and wound healing [57]. This porous structure is most suitable for the treatment of acute wounds [58].

**Figure 6:**
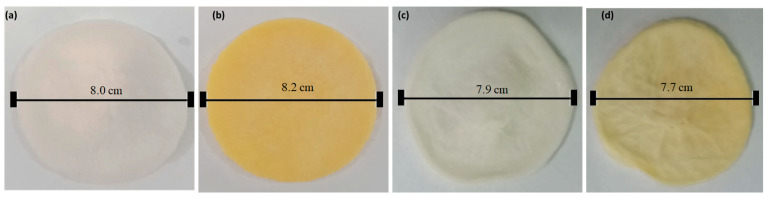
Photographs of hydrogels (**a**) MM-1 (**b**) MM-2 (**c**) MM-3, and (**d**) MM-4 with the scale bar showing the diameter.

The visual/camera images of fabricated hydrogels are shown in Figure 6; the hydrogels’ smooth and uniform texture is visible, indicating their homogeneous nature. MM-1 and MM-3 are clear and white in color, while MM-2 and MM-4 show yellow and light yellowish colors, respectively, due to the LE incorporation.

Hydrogel thickness values vary from 1.39 mm to 1.75 mm. The thickness of MM-1 and MM-2 hydrogels was measured as 1.70 mm and 1.75 mm, respectively, while the thickness of MM-3 and MM-4 hydrogels was noted as 1.39 mm and 1.51 mm. The better thickness values of MM-1 and MM-2, compared to MM-3 and MM-4, can be attributed to higher amounts of PVA in these hydrogels. Similarly, the increased thickness in MM-2 hydrogel (compared to MM-1) and MM-4 hydrogel (compared to MM-3) can be ascribed to extensive crosslinking with LE, which creates a highly interconnected structure [59]. As a result, MM-2 has enhanced water retention capabilities and can effectively absorb moisture, making it more appropriate for wounds that produce exudate.

### 3.2. Crystallinity Patterns

**Figure 7:**
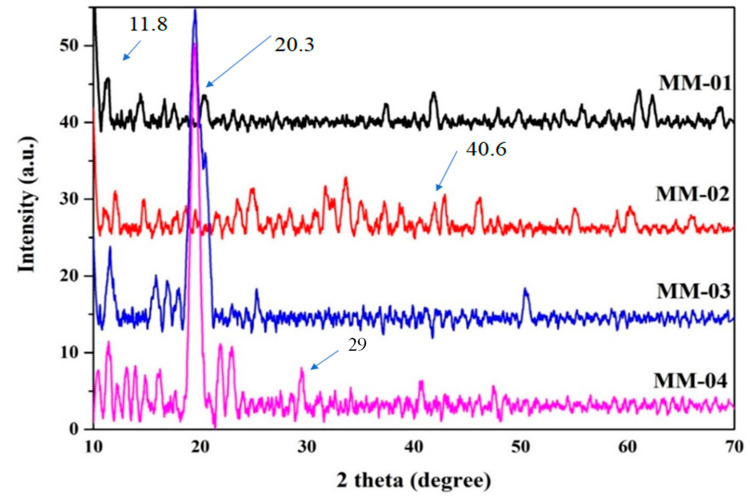
XRD of synthesized CS/PVA/GE/LE hydrogels.

To determine whether the composite hydrogels were crystalline or amorphous in nature, X-ray diffraction (XRD) analysis was conducted. The XRD graphs of all synthesized composite hydrogels are shown in Figure 7. The peaks observed at 2θ = 11° in XRD graph of MM-1 and MM-3 were diminished due to the presence of gelatin, which is amorphous and alters the crystalline structure of PVA. CS typically exhibited a very sharp peak at 2θ = 20.5° in MM-3 and MM-4 hydrogels’ spectra. Another peak with low intensity appeared at 2θ = 40°, which was due to the presence of CS polymer [60].

The hydrogels MM-2 and MM-4 exhibit numerous additional peaks at 2θ = 35° to 35°, which could be attributed to certain compounds present in LE. MM-3 and MM-4 hydrogels displayed a very high intensity peak at 2θ = 20.5°, resulting from the CS, as these hydrogels contain higher amounts of CS. In MM-4, the peak sharpness is more pronounced due to the complete integration of LE flavonoids within the hydrogel structure [61]. These XRD patterns demonstrate the crystalline nature of the interaction between licorice plant extract and CS/PVA/GEL in the MM-4 hydrogel matrix, make it more suitable for deep wounds requiring long-term care [62]. This improvement increases the wound water retention capacity and moisture content, thereby accelerating the healing process [63].

### 3.3. Characterization of Hydrogel Properties for Wound Healing

The hydroxyl groups (OH) in polyphenols are primarily responsible for their ability to detoxify free radicals, making them crucial components of plant extracts. LE’s high phenolic content greatly enhances hydrogels’ antioxidant qualities, which can be extremely important for wound healing and oxidative stress defense. Phenolics from LE facilitate faster and better wound healing by scavenging free radicals, lowering inflammation, and boosting cell proliferation [64]. LE exhibited a total phenolic content of 76.2 ± 1.5 mg GAE/g. These values are close to the already reported literature values by Quintana et al. [65].

A UV–Visible spectrophotometer was used to determine the TFCs of LE by ascertaining that the aluminum ions form a complex with carbonyl and hydroxyl groups of flavonoids. The TFCs of LE were determined as 3.83 ± 0.17 mg of catechins equivalent per g of extract. The determined value is comparable to the already reported value in the literature [66]. Licorice flavonoids, including glabridin, isoliquiritigenin, and liquiritigenin, can aid tissue regeneration by increasing collagen synthesis and fibroblast activity. When hydrogel containing LE is applied as a wound dressing, allowing the phenolics to act precisely at the injury site, where oxidative stress and inflammation are highest, this hastens the healing process [67].

**Figure 8:**
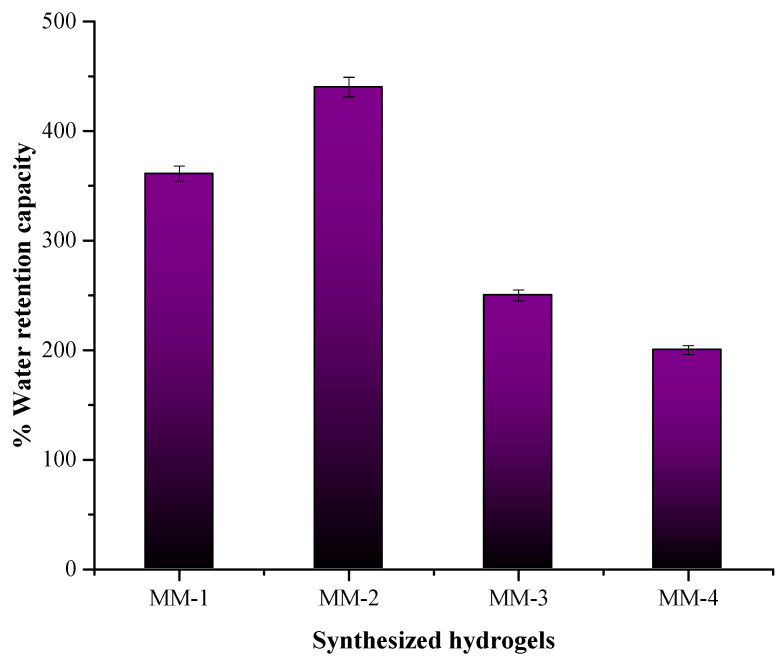
Graph depicting the % water retention capacity in all synthesized hydrogels.

Figure 8 illustrates that MM-1 and MM-2 exhibit the highest water retention capacity. Specifically, MM-2 demonstrates a 440% water retention capacity. The possible reasoning for the highest water retention ability is the presence of PVA (hydrophilic in nature) in a higher ratio, and also many other flavonoids, phenolic acids, and glycyrrhizin (hydrophilic compounds) found in LE. This is especially helpful in establishing a moist environment at the sites of wounds, which is essential for the healing process [68]. In contrast, MM-3 and MM-4 display lower capacities at 250% and 200%, respectively. This difference may be attributed to the reduced quantity of PVA in these hydrogels.

**Figure 9:**
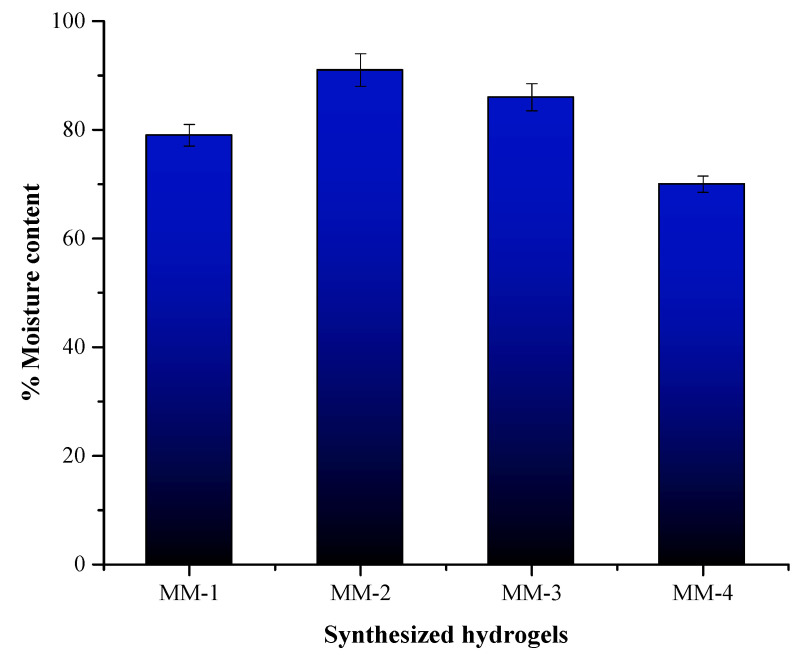
Moisture content (%) in the synthesized hydrogels.

Percent moisture content is a vital parameter for hydrogel performance and is shown in Figure 9. The MM-2 hydrogel demonstrated the highest moisture content percent, i.e., 91%. This high moisture content percent was credited to the presence of a higher ratio of PVA and also due to the incorporation of LE. This feature of hydrogels is important for such wounds that need constant hydration, as it maintains moisture in the wound bed, which ultimately promotes tissue regeneration, cellular migration, growth, and speeds up the healing process. In contrast, MM-4 hydrogels exhibited the lowest percent moisture content, likely due to their reduced PVA content. As illustrated in Figure 9, MM-1 and MM-3 also showed notable percent moisture absorption content, reaching 79% and 86%, respectively.

**Figure 10:**
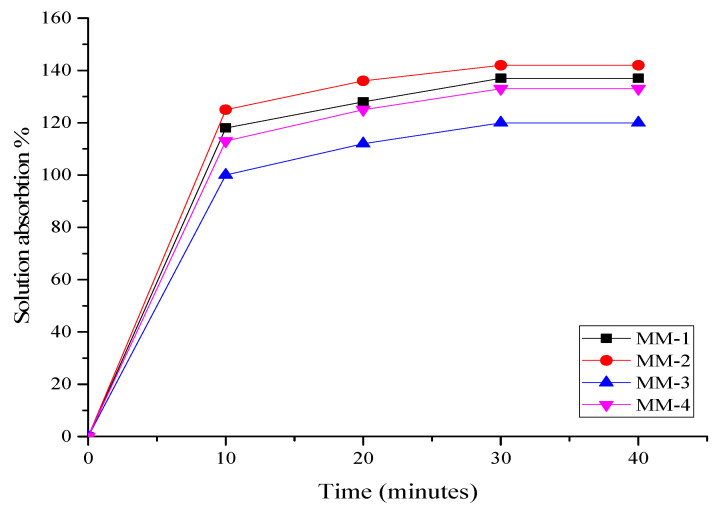
Graphical representation of solution absorption studies of prepared hydrogels.

The synthesized hydrogels were placed in a PBS solution with a pH of 7.4 at 37 °C to assess their percentage of solution absorption. Figure 10 illustrates the findings of this test. The graph indicated that all hydrogels nearly reached their maximum absorption within the first 10 to 20 min, as detailed in Table 3. After 30 min, no additional absorption was observed, and the weight of all hydrogels remained constant. The results revealed that the MM-2 hydrogel absorbed the most solution due to its higher PVA content and porous structure. The hydrophilic properties of PVA make it suitable for absorbing more solution [69]. The synthesized hydrogels MM-1 and MM-2, which contain a higher proportion of PVA, have absorbed the most due to their hydrophilic properties. Similarly, the MM-4 hydrogel absorbed more solution compared to the MM-3 hydrogel. Hydrogels with a greater capacity for solution absorption are more suitable for exuding wounds, as they can manage essential wound fluids and help prevent infections [70].

Oxygen exchange is a crucial component of wound dressings because it promotes cell proliferation and speeds up the healing process [71]. The wound bed contains infections, especially those brought on by anaerobic strains of pathogenic organisms; therefore, oxygen creates a better environment for the healing process. Reduced oxygen concentration penetration on the wound bed slows tissue regeneration or gives anaerobic bacteria a chance to grow stronger [72]. As the results showed, the oxygen permeability measurements of the airtight flask (negative control) and open flask (positive control) were determined to be 4.7 ± 0.1 mg/mL and 5.21 ± 0.1 mg/mL, respectively. The MM-2 exhibited the highest oxygen permeability (5.9 ± 0.1 mg/mL) compared to MM-4 (4.99 ± 0.1 mg/mL). With its increased PVA concentration and licorice extract, MM-2 may produce a more flexible or open network. Because licorice contains a number of hydroxyl groups, the phenolic chemicals may interact with the PVA matrix and form crosslinking. Because of this, the structure becomes more porous, improving the flow of oxygen [73]. Oxygen plays a vital role in various healing processes, including oxidative bacterial elimination, angiogenesis, epithelialization, and the application of wound dressings with adequate O_2_ permeability [74]. In the case of un-crosslinked hydrogels, MM-1 (5.7 ± 0.1 mg/mL) demonstrated better results than MM-3 (5.05 ± 0.1 mg/mL), likely due to its higher PVA content. The oxygen permeability of these hydrogels is within the optimal dressing range.

### 3.4. Biocompatibility and Antibacterial Properties of Hydrogels

**Figure 11:**
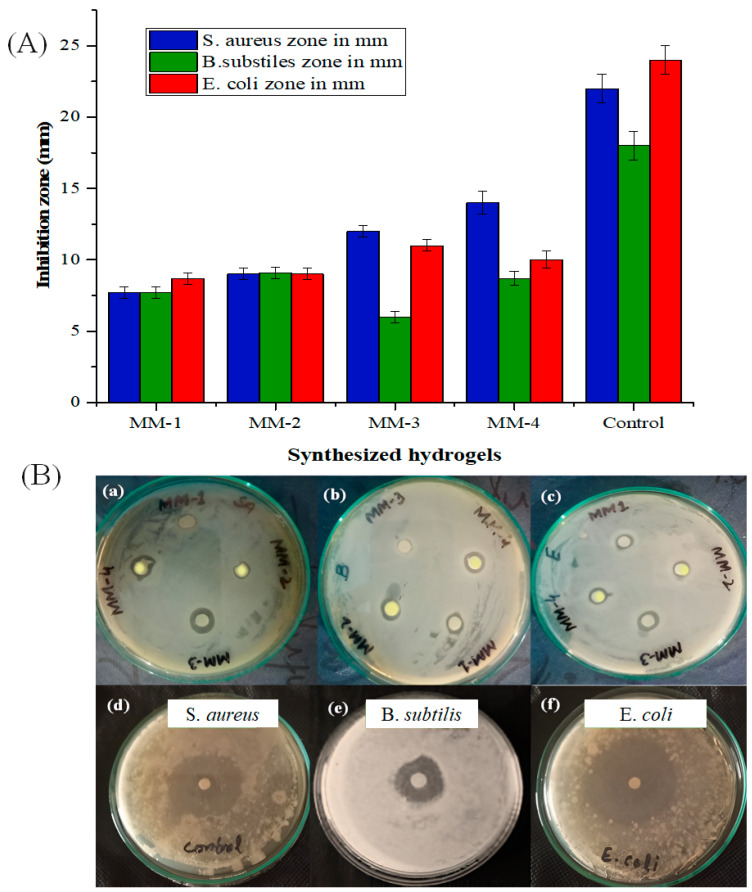
(**A**): Graphical representation of antibacterial activities of all hydrogels and their controls. (**B**): Antibacterial activities of hydrogels (**a**–**c**) in terms of inhibition zone. While control of *S. aureus* (**d**), control of *B. substilis* (**e**) and control of *E. coli* (**f**).

The antimicrobial effectiveness of all the synthesized hydrogels based on CS/PVA/GL, with and without incorporation of LE, was evaluated against three bacterial strains, including gram-positive *B. subtilis*, *S. aureus*, and gram-negative *E. coli*. Figure 11 illustrates the antibacterial activities of all hydrogels.

Amongst all hydrogels, the MM-4 hydrogel showed the highest antibacterial potential against *S. aureus* and *B. subtilis*, which was due to synergistic antibacterial effects of both CS and LE. It is believed that certain biocomponents in LE, including liquiritigenin, glycyrrhizin, isoliquiritigenin and glabridin, have damaged the bacterial cell walls, thus increasing membrane permeability and allowing cellular contents to seep out, ultimately resulting in bacterial cell death. These bioactive components are also believed to work by blocking bacterial DNA gyrase (an enzyme necessary for bacterial replication) to further impede their growth [75].

The MM-3 hydrogel also demonstrated better antibacterial efficacy against *S. aureus*, *B. subtilis*, and *E. coli* strains, likely due to its higher CS content. Chitosan has excellent intrinsic antibacterial potential due to its positive charge in protonated form and presence of active amino and hydroxyl groups, which interact with bacterial cell walls and rupture them to allow internal components to leak out and ultimately result in bacterial cell death. Similarly, when we compared the antibacterial potential of MM-1 with all other hydrogels, it was revealed that MM-1 hydrogel exhibited less antibacterial potential against all bacterial strains. This was mainly due to the absence of LE and also due to the low CS ratio. Thus, it was believed that the incorporation of LE-containing components glycyrrhizin, glabridin, and licochalcone A (MM-2 and MM-4) and increasing the CS ratio (MM-3 and MM-4) significantly enhances antibacterial properties [76]. Literature studies showed that chronic wounds benefit greatly from CS- and LE-based hydrogels due to their exceptional antibacterial activity, low cytotoxicity, and lack of resistance risk [77].

**Figure 12:**
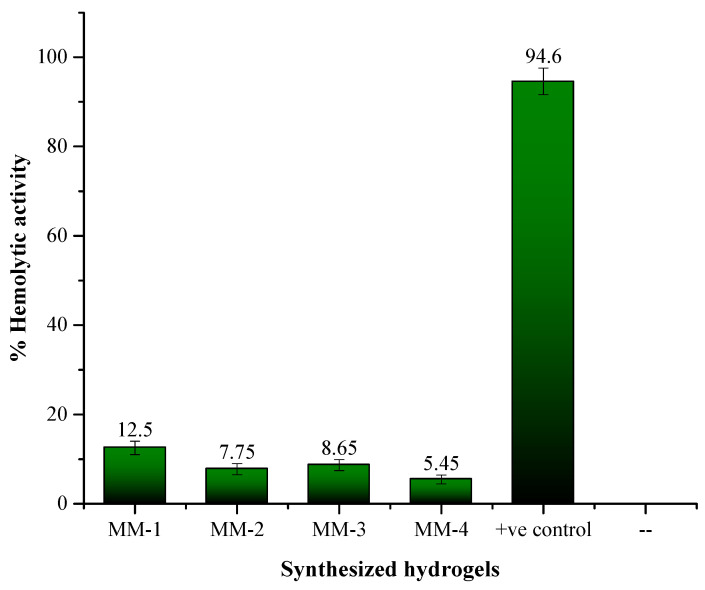
Graphical representation of the hemolytic activity of hydrogels.

A hemolytic assay was conducted on CS/PVA/GEL hydrogels with and without LE, using bovine red blood cells, with results shown in Figure 12. All of the synthesized hydrogels demonstrated non-toxicity to the bovine red blood cells. The negative control (PBS solution, pH = 7.4) showed 0% RBCs lysis, while the positive control (Triton 0.1% X-1000) resulted in 94.6% lysis. Amongst MM-1 and MM-3 hydrogels (without LE), the MM-3 hydrogel showed less cytotoxicity (8.65%) due to the presence of a higher CS ratio. The presence of primary amino groups (-NH_2_) became protonated, or positively charged at physiological pH, thus CS gently interacts with negatively charged biological membrane constituents (such as phospholipids), lowering the possibility of membrane damage [78]. The MM-2 and MM-4 hydrogels (with LE) exhibited the lowest cytotoxicity with 7.75% and 5.45%, respectively. This was mainly due to the presence of flavonoids, glycyrrhizin, and other non-toxic components in LE. These bioactive components preserve cell membranes and reduce oxidative and inflammatory harm, thereby maintaining the integrity of RBCs and ensuring that the hydrogel can be applied to wounds without causing hemolysis. Among the various hydrogels, MM-4 demonstrates the least cytotoxicity due to its higher CS content and presence of LE [79].

### 3.5. Biodegradation Performance

**Figure 13:**
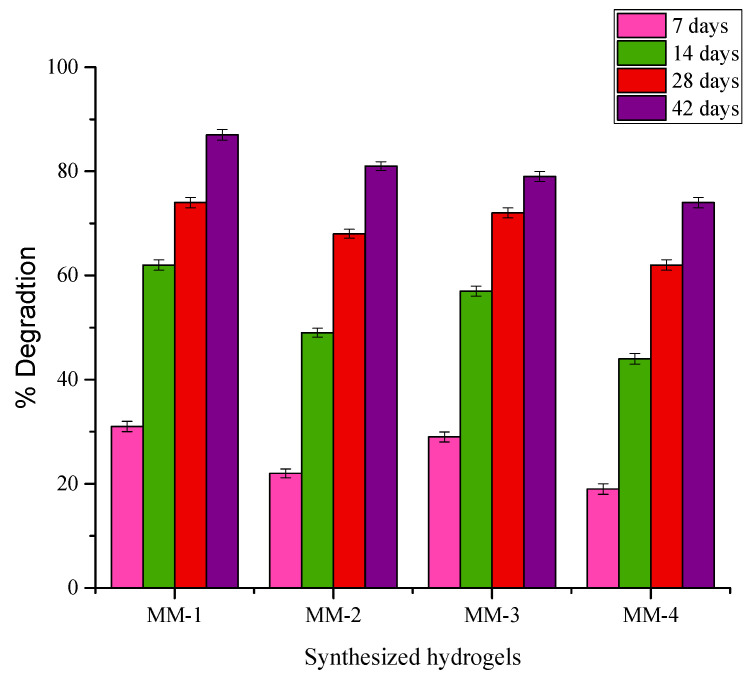
Graphical representation of % biodegradation of synthesized hydrogels.

The hydrogel components, including CS, PVA, and GEL, have low cytotoxicity and are well-known biocompatible materials [80,81], as they do not produce undesirable, harmful byproducts when they break down. The hydrogel’s degradation time should be calculated to correspond with the time needed for new tissue to grow [82]. For better healing or regeneration processes to be successful and seamless, the hydrogel degradation and tissue regeneration processes must be synchronized. Figure 13 shows the % degradation of hydrogels submerged in a PBS solution of pH 7.4 at 37 °C for 42 days. Hydrogels are hydrophilic in nature, and when exposed to PBS solution, water molecules interact with polymer chains by penetrating into the network of polymers, causing the hydrogels to swell. The probability of hydrolytic cleavage rises with increased water absorption [83]. It was noticed that increasing PVA content has increased the % degradation of hydrogels. Thus, the increased PVA ratio in MM-1 and MM-2, as compared to MM-3 and MM-4, has resulted in more degradation of these hydrogels. The hydrophilic nature of PVA encourages more water absorption, and hydrolytic breakdown occurs [84]. Thus, the faster degradation ability of hydrogels is more suitable for acute wound care or burn patients [85]. Similarly, it was also observed that hydrogels with LE (MM-2 and MM-4) degraded less than the hydrogels without LE (MM-1 and MM-3). This might be due to their more interconnected or crosslinked structure because of the presence of LE. As hydrogels degrade slowly, they become more suitable for chronic wound care and maintain a barrier against infections for long periods [86].

### 3.6. Anti-Oxidant Potential of Hydrogels

**Figure 14:**
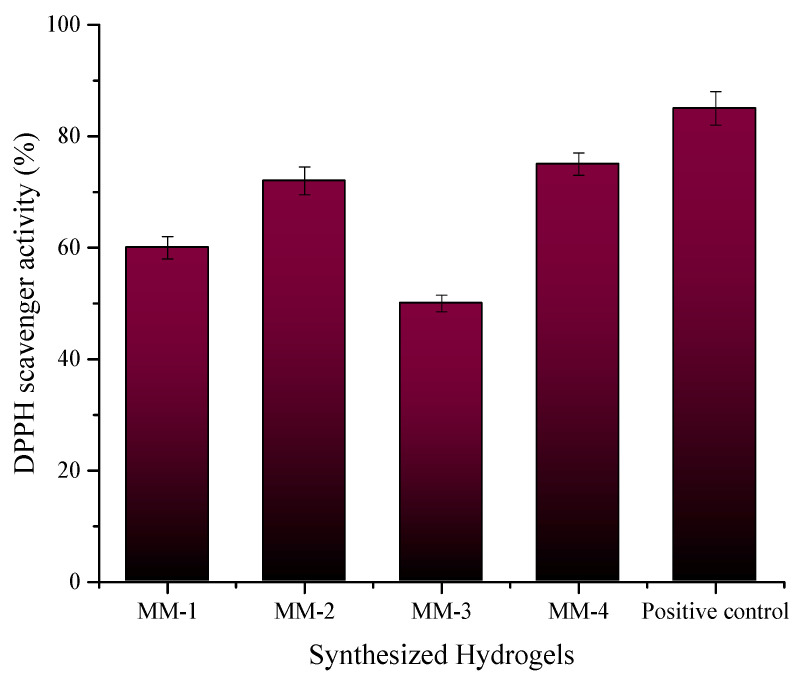
Anti-oxidant potential of MM-1, MM-2, MM-3, MM-4 hydrogels and ascorbic acid as positive control.

Based on the antioxidant activity results, the sample MM-04 demonstrates the highest percentage inhibition (75 ± 0.5%) at a concentration of 1 mg/mL, indicating its strong ability to combat oxidative damage, which is beneficial for chronic wound healing [87], closely followed by MM-02 (72 ± 0.19%) as shown in Figure 14. In contrast, MM-03 shows the lowest inhibition (50 ± 0.15%), indicating weaker antioxidant performance among the tested samples. The sample MM-01 exhibits a moderate inhibition value (60 ± 0.11%). The positive control, ascorbic acid, shows the highest antioxidant activity (85 ± 0.12%), confirming its well-established efficacy as a strong antioxidant.

**Figure 15:**
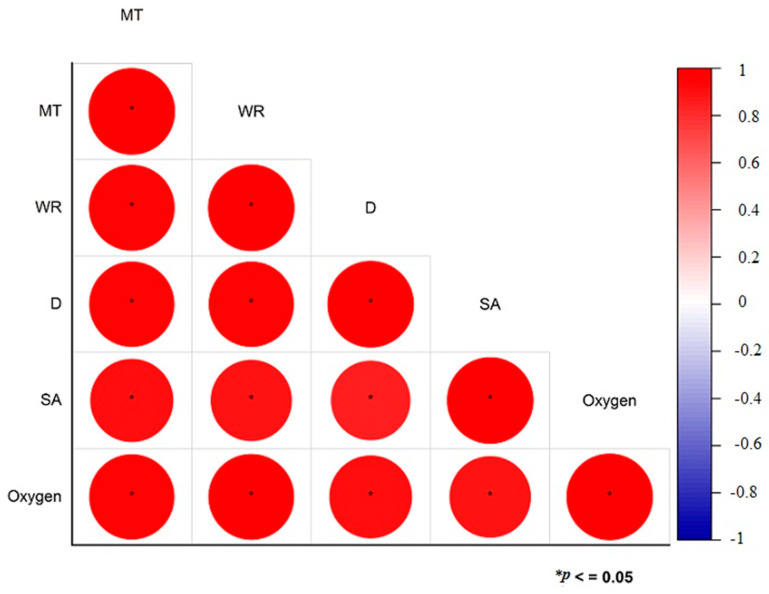
Correlation analysis of MT–WR, WR–D, D–SA, SA–Oxygen.

Correlation coefficients are used to assess the degree and direction of relationships between various variables. The correlation matrix shown in Figure 15 illustrates the connections among different hydrogel parameters that influence wound healing effectiveness. These parameters include matrix thickness (MT), water retention (WR), degradation (D), swelling ability (SA), and oxygen permeability (oxygen). The statistical significance of these correlations is denoted by asterisks (*), with *p*-values of 0.05 or less. A strong positive correlation between these parameters is indicated by the red color in the matrix. As the hydrogels’ matrix thickness increases, there is a corresponding rise in water retention capacity, degradation rate, swelling ability, and oxygen permeability. Other parameters exhibited comparable correlations. The strong positive correlation between MT and WR suggests that hydrogels with enhanced water retention are able to maintain greater matrix thickness, which is crucial for creating a durable and effective wound dressing. The WR–D relationship indicates that hydrogels with higher water retention tend to degrade more quickly, which is beneficial for rapid bioactive release but less ideal for long-term use. Furthermore, the D–SA correlation shows that increased swelling capacity results in faster degradation, emphasizing the balance between swelling and longevity. Lastly, the SA–Oxygen correlation illustrates that greater swelling improves oxygen permeability, which aids in better wound healing. Understanding these correlations allows for the precise adjustment of hydrogel formulations, balancing strength, moisture retention, degradation, and oxygen permeability to cater to specific wound care requirements.

## 4. Conclusions

This study successfully formulated LE-enhanced CS/PVA/GE hydrogels with promising properties for wound dressing applications. The freeze gelation method produced hydrogels with a highly interconnected and porous structure, which demonstrated moisture retention and antibacterial potential. Structural analysis was performed through FTIR, SEM, and XRD, which confirmed effective interactions between various components of the hydrogel matrix. Notably, MM-4 and MM-2 exhibited optimal water retention and oxygen permeability, which are essential for maintaining a moist wound environment and can promote cellular proliferation and tissue regeneration. The inclusion of bioactive LE compounds endowed the hydrogels with robust antibacterial activity against *S. aureus*, *B. subtilis*, and *E. coli*. The hemolysis analysis confirmed their non-toxic nature. These results suggested that synthesized CS/PVA/GEL/LE hydrogels, particularly MM-4, are viable candidates for advanced wound management, combining the benefits of natural extracts with hydrogels to improve wound healing outcomes. Future studies could explore in vivo testing and optimization for clinical applications, reinforcing these hydrogels as reliable solutions in wound care applications. The results of this study were compared with commercial wound dressings such as Aquacel^®^, Acticoat^®^, and Tegaderm^®^. While commercial products often use synthetic antimicrobials like silver, the developed licorice-enhanced hydrogel provides natural antibacterial properties, better biocompatibility, and cost-effectiveness, but requires further standardization and clinical validation for broader use.

## Figures and Tables

**Table 1:** Advantages and limitations of previous studies.

Materials Used	Bioactive Agent	Key Findings	Advantages	Disadvantages	References
Chitosan, Gelatin	Aloe Vera Extract	Effective wound healing and moderate antibacterial properties	Biocompatibility, promotes tissue regeneration	Relatively slow degradation rate	[10]
PVA, Gelatin	Honey	Excellent antibacterial activity	High antibacterial potential due to honey’s properties	Sticky texture and slow degradation rate	[35]
Alginate, Chitosan	Green Tea Extract	Enhanced antioxidant and antibacterial properties	Antioxidant-rich, biocompatible	Limited mechanical strength	[36]
Chitosan, PVA	Curcumin	Strong antibacterial activity, improved healing	Potent antibacterial agent, easy to incorporate	Poor water retention and stability	[37]
Chitosan, PVA, Gelatin	Licorice Extract	Good antibacterial and swelling properties	Natural bioactive agent with antibacterial and wound healing properties	-	This study

**Table 2:** Representation of volume ratios of CS, PVA, GEL and LE solutions in hydrogels.

Sr. No.	Sample Code	CS (mL)	PVA (mL)	GEL (mL)	LE (mL)
1	MM-1	20	20	3	-
2	MM-2	20	20	3	4
3	MM-3	28	12	3	-
4	MM-4	28	12	3	4

**Table 3:** Physiochemical properties of CS/PVA/GEL/LE hydrogels.

Sample Code	Membrane Thickness (mm)	% Water Retention Capacity	% Moisture Content	% Degradation	% Solution Absorption			
					10 min	20 min	30 min	40 min
**MM-1**	1.7	361	79	87	118	128	137	137
**MM-2**	1.75	440	91	81	125	136	142	142
**MM-3**	1.39	250	86	79	100	112	120	120
**MM-4**	1.51	200	70	74	113	125	133	133

Values are the mean of triplicate measurements.

## Data Availability

The raw data supporting the conclusions of this article will be made available by the authors on request.

## Supplementary Materials

The following supporting information can be downloaded at: https://www.mdpi.com/article/10.3390/bioengineering12050439/s1. Figure S1: Homolytic Activity Assay Workflow. Figure S2: Antioxidant potential of the hydrogels and positive control (ascorbic acid). Table S1: Pore size of MM-1. Table S2: Pore size of MM-2. Table S3: Pore size of MM-3. Table S4: Pore size of MM-4. Table S5: Antibacterial Activity. Table S6: Degradation of Hydrogels.
