# Supplementary material for: Formulation and Evaluation of Licorice-Extract-Enhanced Chitosan, PVA, and Gelatin-Derived Hydrogels for Wound Dressing
Source: Bioengineering (Basel). 2025 Apr 23;12(5):439. doi: 10.3390/bioengineering12050439 (PMC12109391; doi:10.3390/bioengineering12050439)
Supplement: Supplementary file 1 [file bioengineering-12-00439-s001.zip › bioengineering-3455303-supplementary.pdf]

Formulation and Evaluation of Licorice-Extract-Enhanced Chitosan, PVA, and Gelatin-Derived Hydrogels for Wound Dressing

Maria Mujahid[a], Muhammad Zubair[b], Asma Yaqoob[c], Sohail Shahzad[a*], Aman Ullah [b*]

[a] *Department of Chemistry, University of Sahiwal, Sahiwal 57000, Pakistan*

[b] *Lipid Chemistry Utilization Lab, Department of Agricultural, Food & Nutritional Science, University of Albert, Edmonton, AB, Canada, T6G 2P5*

[c]*Institute of Biochemistry, Biotechnology and Bioinformatics, The Islamia University of Bahawalpur, Pakistan*

*<u>*Correspondence Authors*</u>*:

Sohail Shahzad: drsohail@uosahiwal.edu.pk

Aman Ullah: ullah2@ualberta.ca

Table S1: Pore size of MM-1

Area	Mean	Min	Max	Angle	Length
17	16.306	11	23.667	7.125	16.055
25	33.907	22.667	61.333	0	8
22	34.18	15.603	50.947	0	11.333
28	36.312	11.489	103	-17.745	26.566
32	59.11	19.57	102.834	-7.352	30.926
33	29.059	16.149	38.99	-26.565	11.609
23	51.307	20.372	86.758	-15.255	2.351
28	27.605	15.7	76	0	10.667
28	36.038	17.52	72	4.236	26.8
38	52.899	14.234	111.396	-137.203	36.782
32	38.513	12	83	-172.648	13.926
12	68.078	52.778	78.667	-109.983	11.392
0.411	24.611	15	53	-87.274	10.777
0.439	36.391	13.769	60	-85.4	2
1.405	15.913	7.564	29.333	-79.824	9.67
1.405	15.913	7.564	29.333	-79.824	9.67
1.405	45.761	17.184	102.111	-81.573	10.106
1.779	26.114	15.097	38.111	-83.48	13.079
0.269	32.749	14.667	64	-94.236	3.72
0.425	24.833	15	51	-90	6.183

Table S2: Pore size of MM-2

Area	Mean	Min	Max	Angle	Length
22	43.709	27.113	74.593	35.218	21.082
33	50.212	18.472	95.667	17.879	32.056
11	72.432	38.133	141.333	36.87	10.414
8	32.611	21.667	60.778	0	6.667
18	40.842	23.431	97.111	0	17.333
34	25.074	3.085	68.444	49.97	33.119
53	33.16	14.367	75.781	164.65	52.392
40	31.522	12.523	64	-120.466	38.873
46	33.386	20.322	71.035	-14.036	45.274
60	21.157	7.114	72.444	45.69	59.404
32	18.981	12.15	25	-132.397	31.127
17	16.288	7	30	180	16
24	18.394	7.206	55	-41.424	22.706
45	21.822	2.394	58.565	-62.85	43.533
16	45.008	20.933	73.593	0	14.667
21	47.746	14.333	84	0	20
16	38.319	14.568	93.667	-3.814	14.727
26	16.842	14.179	24.333	75.964	24.585

Table S3: Pore size of MM-3

Area	Mean	Min	Max	Angle	Length
9241.141	56.199	0	255	0	0
0.887	18.066	10.222	29.099	-68.051	7.916
0.559	36.502	25.084	49.622	-90	4.998
0.571	43.445	24	81.643	-71.175	5.113
0.729	14.621	8	28.464	-38.047	6.498
28	47.184	41.659	53.123	128.991	27.487
31	48.391	32.013	73.044	32.619	29.963
36	25.54	3.657	77.714	-16.858	34.615
27	35.759	15.615	73	100.886	25.889
17	25.23	15	36	-75.964	16.492
9	43.531	27.222	54.333	0	13
9	44.284	28.222	73	90	11
17	52.451	21.333	139	0	16.5
5	39.867	32	48.333	0	6
43	49.632	19.025	99.938	154.654	41.74
1.779	26.114	15.097	38.111	-83.48	13.079
1.732	40.467	28.975	76.667	-86.82	13.509
1.592	27.426	13.071	54.556	-81.439	11.614
56.918	10.58	1	90.333	107.418	82.399
9241.141	56.199	0	255	0	0
0.887	18.066	10.222	29.099	-68.051	7.916

Table S4: Pore size of MM-4

Area	Mean	Min	Max	Angle	Length
0.51	40.827	21.61	75.943	-41.055	4.48
9241.141	56.199	0	255	0	0
0.887	18.066	10.222	29.099	-68.051	7.916
0.559	36.502	25.084	49.622	-90	4.998
0.571	43.445	24	81.643	-71.175	5.113
0.729	14.621	8	28.464	-38.047	6.498
0.644	14.089	8	25.556	1.102	5.735
0.559	25.971	17.071	36.901	-56.659	4.974
0.365	48.252	10.966	106.111	0	3.234
0.51	33.296	13.221	67.809	15.376	4.564
0.413	52.718	12.064	122.778	0	3.675
1.174	18.368	11.991	37.338	-39.472	8.767
1.174	18.368	11.991	37.338	-39.472	8.767
0.981	35.272	25.133	51.927	-31.675	7.295
1.191	40.814	11.838	109.908	-34.641	8.862
0.438	50.459	10.333	100.481	41.634	3.122
1.149	42.515	12.03	94.176	-9.462	8.265
0.519	45.107	15	71	0	3.811
1.297	29.832	19.095	83.969	-35.538	9.367
73	17.471	9	44.988	9.593	71.678
95	19.013	8	61.177	26.565	94.215
75	43.661	18.795	113.361	17.447	73.817
1.568	18.727	4	55.574	-21.718	14.058
79	19.288	9.509	46.603	16.348	78.031
67	53.831	18.679	157.596	9.605	66.198

Table S5: Antibacterial Activity

Inhibition zone against bacterial strains

Sample code	S. *aureus* zone in mm	B. *substiles* zone in mm	E. *coli* zone in mm
MM-1	7.7	7.7	8.7
MM-2	9	9.1	9
MM-3	12	6	11
MM-4	14	8.7	10
Control	24	20	22.6

Table S6: Degradation of Hydrogels

Sample code	7 days	14 days	28 days	42 days
MM-1	31%	62%	74%	87%
MM-2	22%	49%	68%	81%
MM-3	29%	57%	72%	79%
MM-4	19%	44%	62%	74%

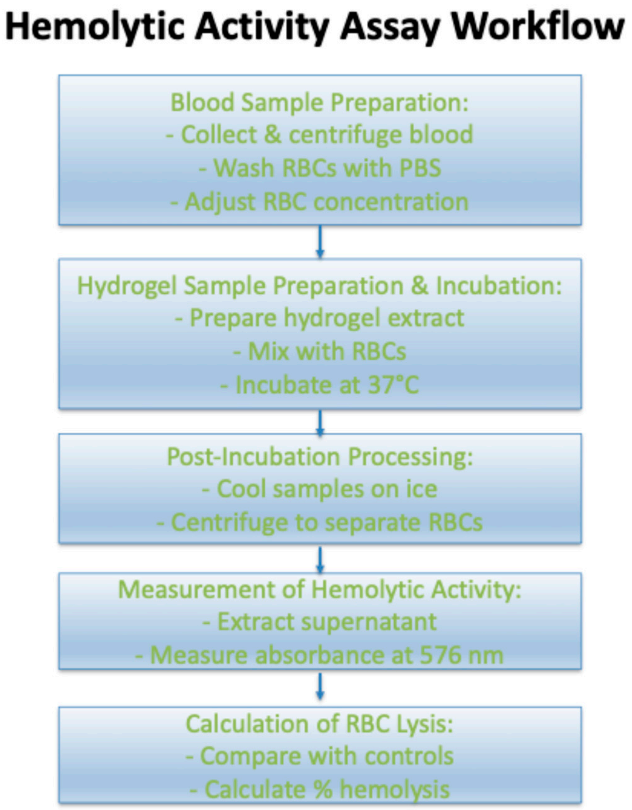

Figure S1: Homolytic Activity Assay Workflow

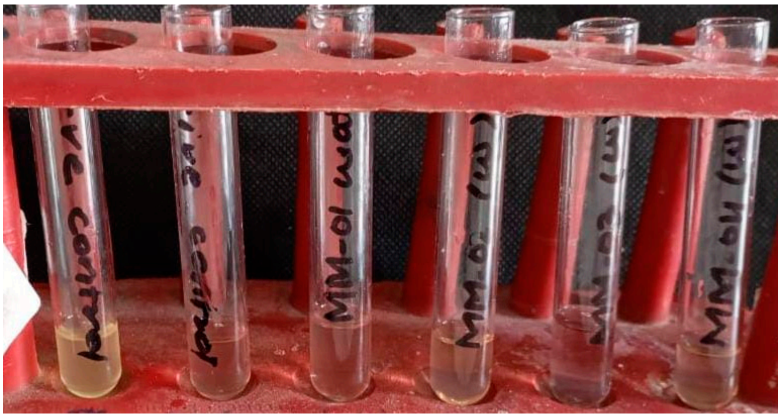

Figure S2: Antioxidant potential of the hydrogels and positive control (ascorbic acid).
